# VEGF-A isoform modulation in an preclinical TNBS model of ulcerative colitis: protective effects of a VEGF164_b_ therapy

**DOI:** 10.1186/1479-5876-11-207

**Published:** 2013-09-11

**Authors:** Walter E Cromer, Chaitanya V Ganta, Mihir Patel, James Traylor, Christopher G Kevil, J Steven Alexander, J Michael Mathis

**Affiliations:** 1 Gene Therapy Program, LSU Health Sciences Center, 1501 Kings Highway, Shreveport, LA 71130-3932, USA; 2 Department of Cellular Biology and Anatomy, LSU Health Sciences Center, Shreveport, LA, USA; 3 Department of Molecular and Cellular Physiology, LSU Health Sciences Center, Shreveport, LA, USA; 4 Department of Pathology, LSU Health Sciences Center, Shreveport, LA, USA

**Keywords:** Ulcerative colitis, VEGF, Angiogenesis, Adenovirus, Therapy, TNBS, Colon, Inflammation

## Abstract

**Background:**

Ulcerative colitis (UC) is the most common form of inflammatory bowel disease in the USA. A key component of UC is the increase in inflammatory angiogenesis of the colon during active disease. This increase is driven to a great extent by the over expression of VEGF-A. Recently, VEGF165_b_ (VEGF164_b_ in mouse), an anti-angiogenic form of VEGF-A was described and its regulation was determined to be disturbed in many pathologies such as cancer and pre-eclampsia.

**Results:**

The aims of this study were to examine the role of this inhibitory VEGF by expressing this molecule in a model of intestinal inflammation, and to evaluate its expression as a potential new therapeutic approach for treating UC. A modified model of TNBS colitis was used to determine the effects of rVEGF164_b_ expression on colon inflammation. Expansion of the vascular system was assessed by immunhistochemical methods and macro- and microscopic measurements of inflammation in the colon were measured. Leukocyte invasion of the tissue was measured by myeloperoxidase assay and identification and counting of lymphoid follicles. Both angio- and lymphangiogenesis were reduced by expression of rVEGF164_b_, which correlated with reduction in both gross and microscopic inflammatory scores. Leukocyte invasion of the tissue was also reduced by rVEGF164_b_ expression.

**Conclusions:**

This is the first report using an endogenous inhibitory VEGF-A isoform for therapy in a model of experimental colitis. Inhibitory VEGF molecules play an important role in maintenance of gut homeostasis and may be dysregulated in UC. The results of this study suggest that restoration of rVEGF164_b_ expression has anti-inflammatory activity in a TNBS model and warrants further examination as a possible therapeutic for UC.

## Introduction

Ulcerative colitis (UC) is a complex intestinal inflammatory disorder that reflects complex interactions between gut tissue, circulating and tissue resident immune cells, gut flora and the vascular system. Data from the National Institute of Diabetes and Digestive and Kidney Diseases (NIDDK) shows that UC is responsible for 244 out of every 100,000 diagnoses and 28 out of every 100,000 hospital admissions. Total cost burden for UC prescriptions is greater than that of Crohn’s disease ($272 million compared with $261 million); this excludes costs for hospital visits [1,2]. Besides the cost of treatment, the impact of UC on quality of life is staggering, and includes increased susceptibility to infection, complications from side effects of therapeutic drugs, and an increased risk for developing colorectal cancer [3-5].

Angiogenesis in the colon during UC results in inflamed vessels that support disease activity and progression through recruitment of immune cells, secretion of inflammatory mediators and increased microvascular and epithelial permeability [6,7]. The increase in the blood vessel density that accompanies UC is predictive of the severity of disease. Inhibition of intestinal microvasculature expansion in UC has been show to be protective against experimental colitis [8,9]. VEGF-A, a key mediator of angiogenesis in UC, has been found to be up regulated in the sera of UC patients and in animals with experimental UC [10,11]. Experiments in animal models of UC have shown that inhibiting VEGF-A signaling can attenuate disease activity while increased levels of VEGF-A exacerbate disease severity [9,12]. Further evidence from models of inflammatory angiogenesis in the eye and more recent models of UC have identified VEGF164 (165 in humans) as the major pathological isoform of VEGF-A. This evidence implicates VEGF164 driving inflammation through altered vascular tone, leakage, remodeling, leukocyte recruitment and changes in the sub endothelial extracellular matrix [13].

We have previously reported the *in vitro* ability of the murine homologue of human VEGF165b, known as VEGF164_b_ (the main endogenous anti-angiogenic VEGF-A isoform), to inhibit endothelial cell proliferation, migration and changes in vessel permeability caused by VEGF-A [14]. Based on our results, we examined the potential therapeutic application of this endogenous inhibitor of VEGF-A signaling in an *in vivo* model of experimental UC. We hypothesized that since rVEGF164_b_ should block blood vessel formation and reduce disease activity by interfering with VEGF-A signaling in our model of UC that it would also diminish evidence of inflammatory injury. We found that rVEGF164_b_ attenuated disease activity, reduced blood and lymphatic vessel formation, and inhibited several markers of disease. Our findings suggest that shifts in VEGF isoform expression may regulate UC activity, indicating potential applications of the VEGF164_b_ isoform as a therapeutic for UC.

## Material and methods

### Virus construction

As previously described [14], a 570 base pair coding sequence of mouse VEGF164_b_ cDNA was synthesized (GeneScript; Piscataway, NJ) to omit a single arginine residue at the COOH terminal of the murine VEGF164_b_ resulting in rVEGF164_b_. The rVEGF164_b_ cDNA was sub cloned into the adenovirus shuttle vector pAdenoVator CMV5 (MP Biomedicals; Solon, OH). A shuttle vector containing the rVEGF164_b_ coding sequence was linearized with PacI, and co-transformed with pAdenoVator ΔE1/E3 (containing the adenovirus backbone sequence) into the *E. coli* strain BJ5183 for homologous recombination. After isolation of recombinants from positive clones, an adenovirus vector Ad5-CMV-rVEGF164_b_, was rescued by transfection of PacI linearized plasmid DNA into the HEK293 packaging cell line.

### Induction of TNBS colitis

All procedures were approved by the LSUHSC Institutional Animal Care and Use Committee (IACUC) under NIH and USDA guidelines. TNBS colitis was induced using a modified version of the protocol of Morris *et al.*[15]. In this model, BALB/c mice were anaesthetized by intraperitoneal injection of Ketamine/Xylazine and were checked for sedation by toe pinch stimulation. Ethanol (100 μL of 10% v/v in water) containing 2 mg of TNBS (Sigma-Aldrich; St. Louis, MO) was slowly introduced into the colon using a rubber tipped gavage needle inserted 5 cm from the anus. Animals receiving vehicle alone (10% ethanol) were dosed in the same fashion. TNBS and vehicle were administered every seventh day resulting in a cyclical disease pattern. The Ad5-CMV-rVEGF164_b_ (1 × 10^11^ viral particles) was administered once daily for three days before the induction of TNBS colitis to maximize effect of the recombinant protein. Animals were euthanized at day 3 (acute phase; n = 5) and day 21 (chronic phase; n = 5).

### Measurement of VEGF-A isoforms

Serum and tissue levels of VEGF-A isoforms were measured by mouse VEGF-A ELISA (R&D Systems; Minneapolis, MN) in both the TNBS (BALB/c) and DSS (C57BL/6) models of UC at days 7 and 10. Serum levels of VEGF-A isoforms were measured using 5 μL of serum collected in serum separator tubes (BD; Franklin Lakes, NJ). Tissues were homogenized in ice cold PBS containing protease inhibitors (Sigma-Aldrich) followed by removal of the insoluble fraction by centrifugation at 10,000 × g for 15 min. Total protein level was adjusted to load a final concentration of 10 ng/well.

### Measurement of occult blood, stool form and weight loss to determine disease activity

Disease activity index is defined as a composite score of severity in weight loss, presence of occult blood in stool, and aberrant stool form. The score for each category is added together and divided by 3, which generates an average index of the contribution of each aspect of disease to overall score. Occult blood was measured by applying a thin smear of fresh fecal matter to a ColoScreen test card (Helena Laboratories; Beaumont, TX) and adding a drop of color change reagent. Fecal blood levels were graded on a 0–4 scale based on the following color changes: 0, no change; 1, slight change (random light blue speckled pattern); 2, moderate change (solid light blue smeared pattern); 3, severe change (solid dark blue smeared pattern); and 4, surface blood. Stool form was measured by grading stool shape and deformation under pressure. Stool was graded using a 0–4 scale based on: 0, fully formed dry stool; 1, fully formed moist stool; 2, fully formed soft stool; 3, unformed soft stool; and 4, diarrhea. These scores were summed with weight change (1, > −5% weight change; 2, > −10% weight change; 3, > −15% weight change; 4, < −20% weight change) and divided by 3 to create a weighted disease activity index score.

### Animal organ harvest

After the animals were euthanized, tissue from the colon to the cecum was removed. The tissue lengths were measured from the junction of the cecum and colon to the anus, afterwards the cecum and feces were removed from the colons and the weights were determined. A length to weight ratio was calculated as colon weight (in mg) divided by colon length (in cm). The colons were divided into 4 sections, and the most distal section fixed in 1% paraformaldehyde. The remaining 3 sections were frozen for further processing. Livers, kidneys, lungs and spleens were removed, weighed, and examined my histopathological analysis to gauge any adenovirus-mediated inflammation.

### MPO assay

The proximal most sections of the colon were homogenized in HETAB buffer, subjected to 3 freeze thaw cycles, and adjusted to a protein concentration of 20 mg/mL. The homogenates were centrifuged at 10,000 × g for 15 min and the supernatants were used for the MPO assay at 50 ng/μL total protein per well. Protein samples were incubated with 150 μL of 0.17 mg/mL o-dianisidine suspended in potassium phosphate buffer pH 6.0 with 0.001% H_2_O_2_. Color change was measured after 20 min at 470 nm on a MCC340 microplate reader (Finstruments; Helsinki, Finland).

### Protein content of TNBS colitis serum

Blood samples were harvested at days 3 and 21. Serum was separated by centrifugation in serum separator tubes (BD) at 8,000 rpm for 90 sec followed by diluted to a working concentration with water (10×). Protein content was measured using a Bio-Rad DC protein assay kit following the protocol for microplates. Data was normalized to control and significance was determined by ANOVA with Dunnett’s post test.

### Histopathology/Measurements

Histopathology was measured by blinded scorers based on area involved in inflammation (0–4), crypt damage and loss (0–4), edema (0–3), follicular aggregates (0–3), surface erosion (0–3) and mononuclear cell and polymorphonuclear cell infiltration (0–3). Scores for each category were combined to give an overall histopathology score. In addition, total follicle aggregates containing a mixture of lymphocytes and leukocytes including macrophages were identified by histopathology and counted. Sections were stained by immunohistochemistry for Mac-2 (Accurate Chemicals, Westbury, NY) to confirm the structures contained macrophages.

### Blood vessel/Lymph vessel staining and counting

Slides of distal colon were deparaffinized in xylene and graded alcohol and followed by antigen retrieval in citrate buffer under steam for 20 min followed by defixation in 0.01% sodium borohydride for 20 min and were peroxide quenched in 90% methanol containing 3% H_2_O_2_ for 20 min. Sections for counting were blocked in 10% goat serum in PBS for 1 h followed by incubation in primary antibody for blood vessels at 1:200 anti-MECA32 or lymphatic vessels 1:200 anti-VEGFR-3 (eBioscience; San Diego, CA) diluted in antibody amplifying dilution buffer (PRO Histo; Columbia, SC) overnight at 4°C. Slides were washed in antibody amplifying wash buffer (PRO Histo) followed by incubation in a biotin conjugated secondary cocktail for 2 h and washed and then incubated in streptavidin HRP for 30 min and washed. Slides were developed using DAB (Dako; Glostrup, Denmark) and counterstained with hematoxylin.

### Vessel counting

Vessels (blood and lymphatic) were counted in 3 undamaged colon sections per slide that had been stained for MECA-32 (blood vessels) and VEGFR3 (lymphatic vessels) at 40× magnification on an Olympus BX50 microscope (Olympus; Center Valley, PA). The same procedure was carried out for both acute and chronic samples by a blinded counter and the resulting data was analyzed by ANOVA.

### Statistical analysis

Statistics were performed using the Instat software package (Scottsdale, AZ). All data was analyzed by ANOVA with Dunnett’s post testing to determine significant difference (p < 0.05) from control. A two-tailed Student’s t test was performed on data to determine significant difference (p < 0.05) between VEGF164_b_ treated and untreated animals.

## Results

### Measurement of VEGF serum levels in TNBS and DSS models of colitis

**Figure 1:**
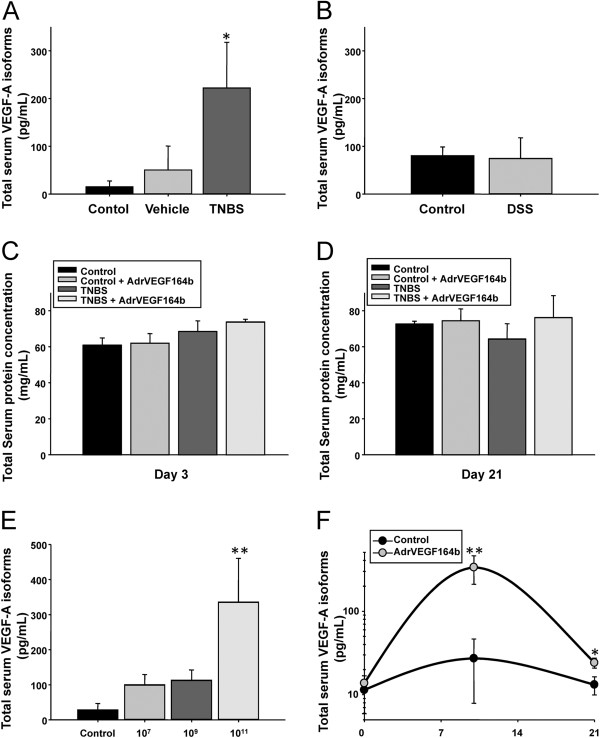
**Measurement of serum levels of VEGF in the TNBS and DSS models of UC.** The level of total serum VEGF-A isoforms was measured by ELISA in serum from control, vehicle, and TNBS treated mice **(A)**; n = 4. The level of total serum VEGF-A isoforms was measured by ELISA in serum from control and DSS treated mice **(B)**; n = 4. Serum protein was measured at either the acute **(C)** or chronic stages of the model **(D)**; n = 5. Levels of total serum VEGF-A isoforms was measured in serum of animals treated with Ad5-CMV-rVEGF164b at 7 days after injection of Ad5-CMV-rVEGF164_b_ using doses of 0 (control), 1 × 10^7^, 1 × 10^9^, and 1 × 10^11^ viral particles **(E)**. An estimate of the persistence of expression of rVEGF164_b_ was generated by measuring total serum VEGF-A isoforms in the serum of animals injected with 1 × 10^11^ viral particles of Ad5-CMV-rVEGF164_b_ at days 0, 10, and 21 **(F)**.

Increased serum VEGF-A has been documented in active human UC and in many animal models of UC. Thus, we determined serum levels of VEGF-A in both TNBS and DSS models of UC to determine which model could best represent human UC. Using a VEGF ELISA we found that our model of TNBS colitis had significantly elevated VEGF-A in the serum (16.2-fold) compared with vehicle-treated animals (Figure 1A). In contrast, we could not detect a statistical difference in serum VEGF-A levels between control and DSS treated mice (Figure 1B). Interestingly, there was also a strain difference in basal VEGF-A expression levels between C57BL/6 mice and BALB/c mice, with 5.8-fold more VEGF-A in the serum of control C57BL/6 mice (74.09 ± 19.82 pg/mL) compared with BALB/c mice (13.70 ± 0.01 pg/mL). These data agree with work by Chidlow *et. al.*[11], suggesting the TNBS model of UC was most appropriate for our study. Another important clinical marker of UC is developing protein losing enteropathy (PLE), representing the loss of serum protein content into the intestine. In our model of TNBS colitis using 10% ethanol we did not observe significant protein loss when measuring total protein levels of serum at either acute (Figure 1C) or chronic (Figure 1D) time points. This result may reflect a different form of injury or severity of injury compared with the DSS or other models of UC.

### Expression VEGF in TNBS and DSS models of colitis

We previously characterized the ability of VEGF164_b_ to inhibit endothelial cell proliferation, migration and changes in vessel permeability *in vitro* caused by VEGF-A [14]. We generated an adenoviral vector (Ad5-CMV-rVEGF164b) expressing a murine recombinant VEGF164b (rVEGF164b) and showed that rVEGF164b blocks the effect of VEGF-A to stimulated proliferation, barrier dysregulation and cytoskeletal rearrangement of murine microvascular endothelial cells (MVECs). These findings are significant since these effects could result in inhibiting new vessel formation and vessel leakage *in vivo*. Excessive vessel formation and permeability are key markers of inflammation, and correlate to the pathogenesis of many diseases including UC. Based on these *in vitro* results we examined the potential therapeutic application of this endogenous inhibitor of VEGF-A signaling in a model of experimental UC *in vivo*. Currently, no specific antibodies are available that can distinguish the VEGF164b isoform of VEGF, from endogenous VEGF-A. Since VEGF-A and rVEGF164b are both bound by anti-VEGF-A we estimated the change in rVEGF164b production in response to adenovirus based on the increase in total VEGF-A signal which occurred in response to treatment with Ad5-CMV-rVEGF164b. In this experiment, we measured total VEGF isoforms by ELISA in serum of animals treated with Ad5-CMV-rVEGF164b at 7 days after injection using doses of 1 × 10^7^, 1 × 10^9^ and 1 × 10^11^ viral particles (Figure 1E*)*. This analysis showed a significant increase in total VEGF-A isoforms in mice administered 1 × 10^11^ viral particles of Ad5-CMV-rVEGF164b compared with control (untreated) mice. In addition, we generated an estimate of persistence in rVEGF164_b_ expression in the serum of animals injected with 1 × 10^11^ viral particles at days 0, 10, and 21 (Figure 1F*)*. This was only an estimation of rVEGF164_b_ expression, determined by comparing total VEGF-A isoforms in serum from Ad5-CMV-rVEGF164b treated mice to total VEGF-A isoforms in serum from mice treated with an equivalent dose of a control vector (Ad5-CMV-GFP). While mice treated with Ad5-CMV-GFP showed no significant change in total VEGF-A isoforms, mice treated with Ad5-CMV-rVEGF164b showed a transient increased expression. This increase was highest at day 10 and was decreased by day 21, a result typical of a non-replicative/non-integrating adenovirus expression vector.

### Assessment of DAI

**Figure 2:**
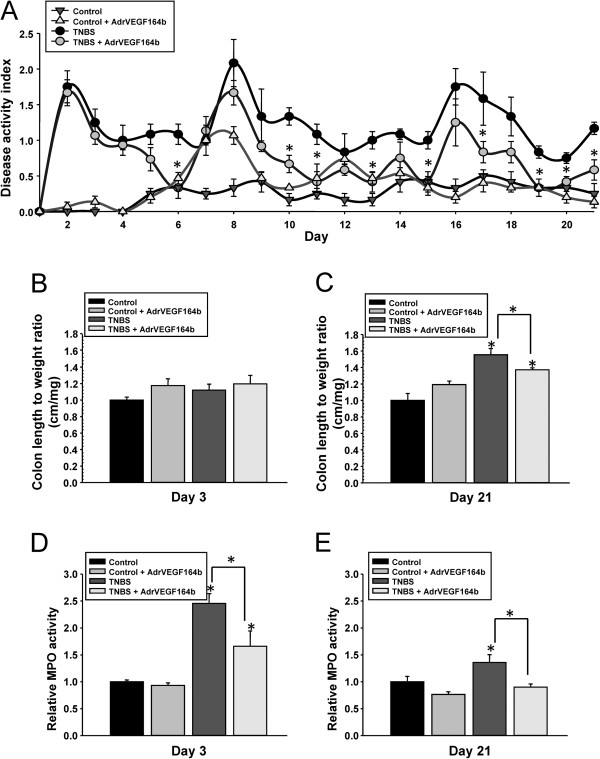
**Assessment of disease progression in the TNBS model of UC.** Disease activity index (DAI) was determined in control, Ad5-CMV-rVEGF164_b_, TNBS, and TNBS + Ad5-CMV-rVEGF164_b_ treated mice. * denotes significant differences between treated and untreated TNBS mice on that day. **(A)**; n = 5. Colon length to weight ratio was determined in control, Ad5-CMV-rVEGF164_b_, TNBS, and TNBS + Ad5-CMV-rVEGF164_b_ mice at day 3 **(B)** and day 21 **(C)** of disease. MPO assay results at day 3 **(D)** and day 21 **(E)** in disease were determined from control, Ad5-CMV-rVEGF164_b_, TNBS, and TNBS + Ad5-CMV-rVEGF164b colons; n = 5. Shown are *p < 0.05, **p < 0.005.

We initially examined disease activity index (DAI), a composite score of severity in weight loss, presence of occult blood in stool, and aberrant stool form in control mice, mice treated with Ad5-CMV-rVEGF164_b_, in TNBS mice, and in TNBS mice treated with Ad5-CMV-rVEGF164_b_ (Figure 2A). TNBS treatment resulted in significant elevation of DAI after day 1 that lasted the duration of the experiment (21 days). Most of the increase in DAI scores in TNBS treated mice was due to elevated occult blood scores and stool form scores with minimal alterations in animal weight (data not shown). Control mice that received Ad5-CMV-rVEGF164_b_ alone had no significant changes in DAI. However, TNBS mice that received Ad5-CMV-rVEGF164_b_ showed significant protection at several days (including days 6, 10, 11, 13, 15, 17 and 19–21) as determined by Student’s t-test versus untreated TNBS mice (Figure 2A). The Ad5-CMV-rVEGF164_b_ treated TNBS mice also recovered faster after each subsequent TNBS injection compared with untreated TNBS mice. These data suggest that reduced VEGF-A signaling by treating mice with Ad5-CMV-rVEGF164_b_ inhibited the TNBS mediated mechanisms that sustain inflammation.

### Determination of colon length, weight, and LXW ratio

As colitis progresses, the length of the colon becomes shorter and it becomes thicker in circumference as the muscles of the colon contract. These factors serve as clinical markers for the progression of chronic inflammation in UC [16]. The ratio of the colon length and weight (LXW) is commonly used to determine levels of inflammation in models of UC. In the TNBS model at day 3 we found no significant alterations in either colon length or weight (Figure 2B) while at day 21 we found that TNBS increased the colon LXW score (55.4% ± 7.7%) compared with control. Interestingly, rVEGF164_b_ treatment reduced the LXW significantly in TNBS mice to 37.2% ± 2.3% above control scores (Figure 2C). The reduction of LXW ratio score in TNBS shows that inhibiting VEGF-A signaling by rVEGF164_b_ treatment can effect gross, clinically relevant measurements of chronic inflammation in the colon.

### Immune cell infiltration in the colon

MPO activity was used as an indirect measurement of neutrophil infiltration in the colon, which is an important measurement of acute inflammation in the tissue. We observed that at day 3, the rVEGF164_b_ treatment significantly reduced MPO activity in the colons of TNBS mice compared with untreated TNBS mice, while treatment had no effect on the MPO level in control animals (Figure 2D). Interestingly, the MPO activity at day 21 in TNBS mice treated with rVEGF164_b_ was reduced to levels in control mice. However, the MPO activity in untreated TNBS animals remained significantly elevated compared with control levels (Figure 2E). These results suggest that rVEGF164_b_ treatment has effects on acute as well as chronic markers of inflammation in our model of TNBS colitis.

**Figure 3:**
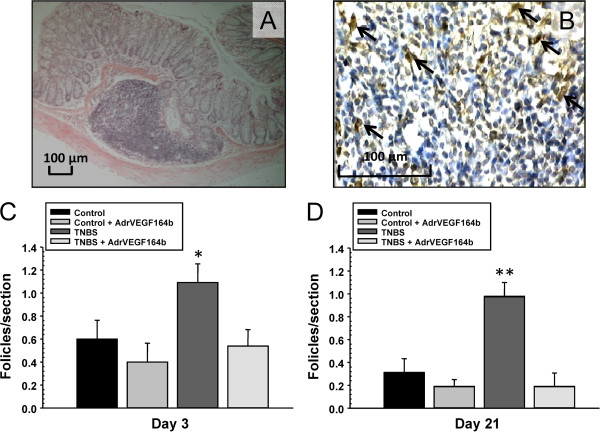
**Determination of lymphoid follicle aggregates in the TNBS model of UC.** Representative image (10× magnification) shows detailed composition of follicle aggregate in TNBS treated colon **(A)**. Representative image (40× magnification) of follicle aggregate showing staining for Mac-2 positive cells (arrows) **(B)**. The number of follicles were quantified at day 3 **(C)** and 21 days **(D)** of disease; n = 5.

In sections of colon we identified follicle aggregates containing a mixture of lymphocytes and leukocytes by histopathological examination (Figure 3A). These sections were immunostained for expression of macrophage subpopulation-specific antigen (Mac-2) to confirm the structures contained macrophages (Figure 3B). Interestingly, the elevation in the number of follicle aggregates (which are a sign of increased immune activity in the colon) induced by TNBS treatment was reduced by rVEGF164_b_ treatment to control levels at both the acute (Figure 3C) and chronic (Figure 3D) phases of TNBS [17].

### Evaluation of blood vessel/lymphatic vessel density

**Figure 4:**
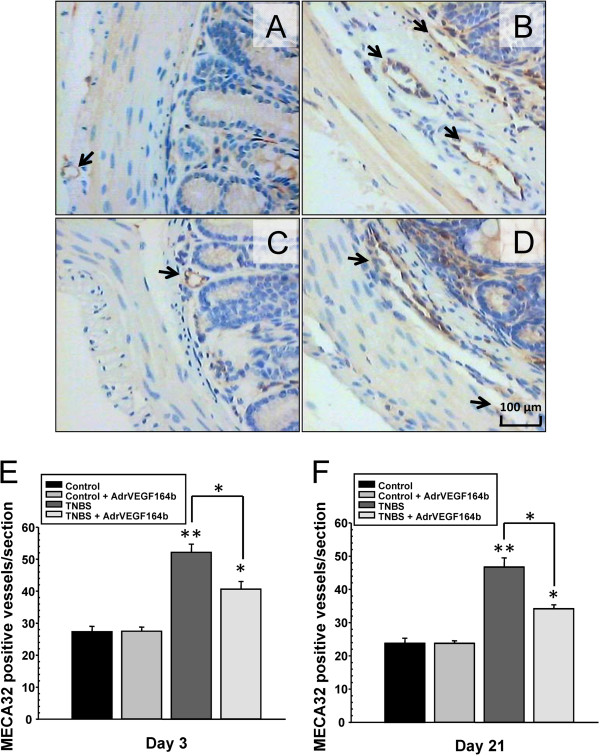
**Evaluation of blood vessel density in the TNBS model of UC.** Representative images of colon sections (20× magnification) are shown from control **(A)**, TNBS **(B)**, Ad5-CMV-rVEGF164_b_**(C)** and TNBS + Ad5-CMV-rVEGF164_b_**(D)** mice immunostained with stained with MECA-32. Angiogenesis was quantified at day 3 **(E)** and day 21 **(F)** of disease; n = 5.

The reduction in inflammatory scores may be explained by inhibiting blood vessel formation. This inhibition in turn, could reduce the area of inflamed and permissive endothelium available to recruit cells from the circulation as well as reduce blood vessel permeability leading to less loss of fluid from the vessels into the adjacent tissue. To quantify changes in blood vessel density, we stained slides of colon sections from untreated control mice (Figure 4A), TNBS treated mice (Figure 4B), Ad5-CMV-rVEGF164_b_ treated mice (Figure 4C), and TNBS + Ad5-CMV-rVEGF164_b_ treated mice (Figure 4D) with antibodies against a marker of blood vessels (MECA32). Subsequently, we counted the number of positively staining blood vessels in each section. The number of blood vessels per section of colon in TNBS mice increased significantly to 2.0-fold compared with control mice as early as day 3 (Figure 4E) during the acute phase of inflammation and was maintained at 2.1-fold higher over control mice on day 21 (Figure 4F). The rVEGF164_b_ treatment did not completely block angiogenesis, but it did reduce angiogenesis significantly at both time points in TNBS mice. This result correlates with data on colon LXW, MPO and histopathology, suggesting that while angiogenesis may be one of many driving factors in UC, it is responsible for increased tissue area affected, immune cell infiltration, and disease severity that make up the complex etiology of UC.

**Figure 5:**
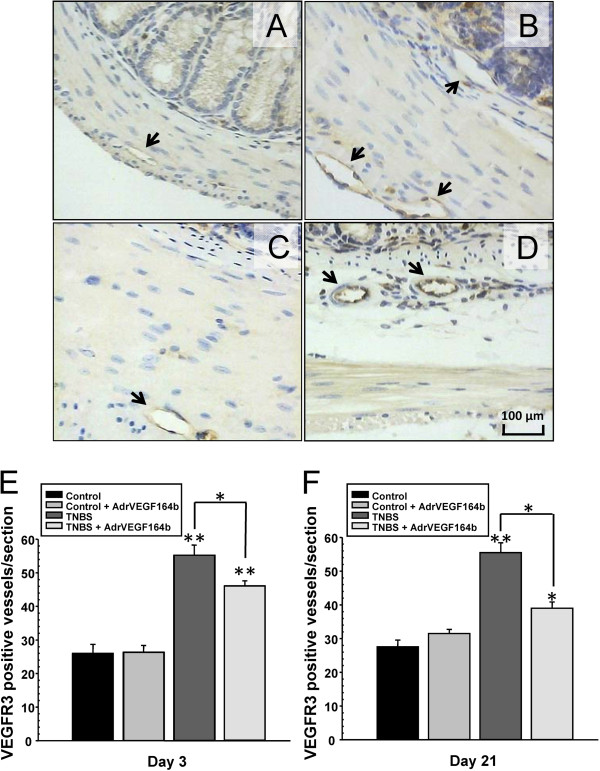
**Evaluation of lymphatic vessel density in the TNBS model of UC.** Representative images of colon sections (20× magnification) are shown from control **(A)**, TNBS **(B)**, Ad5-CMV-rVEGF164_b_**(C)** and TNBS + Ad5-CMV-rVEGF164_b_**(D)** mice immunostained with VEGFR-3. Lymphangiogenesis was quantified at day 3 **(E)** and day 21 **(F)** of disease; n = 5.

To quantify changes in lymphatic vessel density, we stained slides of colon sections from untreated control mice (Figure 5A), TNBS treated mice (Figure 5B), Ad5-CMV-rVEGF164_b_ treated mice (Figure 5C), and TNBS + Ad5-CMV-rVEGF164_b_ treated mice (Figure 5D) with antibodies against markers of lymphatic vessels (VEGFR-3). Afterwards, we quantified the number of positively staining lymphatic vessels in each section. In the stained sections, we observed a decrease in lymphangiogenesis by rVEGF164_b_ treatment in TNBS mice (Figure 5E and 5F), which was proportional to the decrease in angiogenesis. This result may suggest a direct link between angiogenesis and lymphangiogenesis in the TNBS model of UC.

### Analysis of histopathology changes

**Figure 6:**
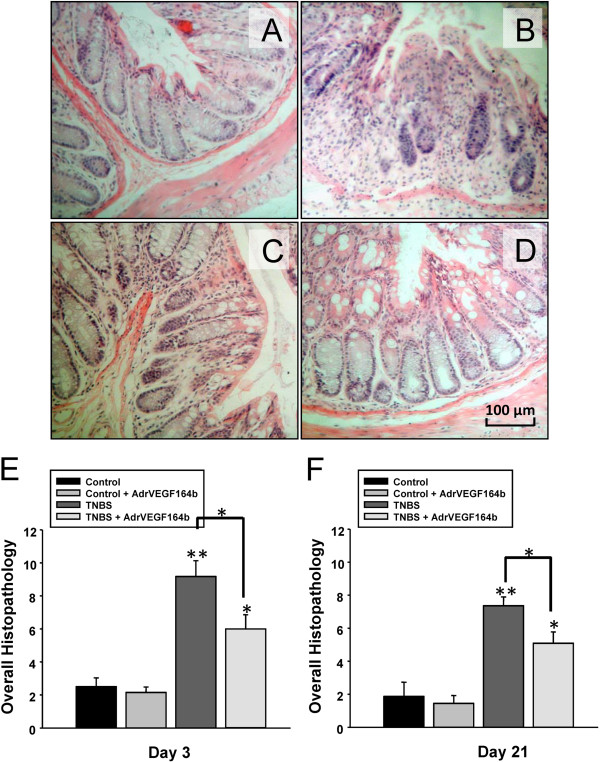
**Analysis of histopathology changes in the TNBS model of UC.** Histopathology was scored by 2 blinded scorers on 6 parameters. Representative images of hematoxylin and eosin stained sections (20× magnification) are shown from control **(A)**, TNBS **(B)**, Ad5-CMV-rVEGF164_b_**(C)** and TNBS + Ad5-CMV-rVEGF164_b_**(D)** treated mice. Histopathology scores for colons on day 3 **(E)** and day 21 **(F)** of disease were combined from both blinded scorers; n = 5.

We examined the effect of rVEGF164_b_ treatment on histopathologic changes in disease progression using the TNBS model of UC, by comparing untreated control mice (Figure 6A), TNBS treated mice (Figure 6B), Ad5-CMV-rVEGF164_b_ treated mice (Figure 6C), and TNBS + Ad5-CMV-rVEGF164_b_ treated mice (Figure 6D). We quantified the histopathologic changes as overall histopathology scores; these data showed the TNBS mice scored significantly higher than control mice in edema, inflammatory cell infiltration and area involved (Figure 6E and 6F). However, there was significant protection with Ad5-CMV-rVEGF164_b_ treatment in TNBS induced UC at both the acute and chronic phases of inflammation.

## Discussion

Angiogenesis occurring under pathological conditions such as UC often results in inflammation driven by newly formed and remodeled blood vessels. We hypothesized that inhibition of VEGF-A signaling using the endogenous inhibitor of VEGF-A signaling VEGF164_b_ would reduce injury resulting from inflammatory angiogenesis. In this study we demonstrated significant protection from TNBS induced colitis in VEGF164_b_ treated animals. In the normal human colon the level of inhibitory VEGFs are 20 fold higher than pro-angiogenic VEGFs suggesting over expression of VEGF164_b_ in UC may restore a normal balance of VEGF-A mediated signaling [18]. In this study, we demonstrated VEGF164_b_, the endogenous inhibitor of VEGF-A signaling, is an ideal candidate for exploring the role of this class of inhibitory VEGFs in the etiology of UC and for possible therapeutic use.

TNBS treatment significantly elevated serum VEGF-A levels compared with the DSS model of UC, which has no difference in serum VEGF-A levels compared with control mice. Interestingly, Chidlow *et al.* found no elevation of VEGF-A levels in the DSS model of UC [9,11,12]. In contrast, Scaldeferi *et al.* and Danese *et al.* suggested that VEGF-A plays a pivotal role in DSS colitis [9,11,12]. We found that tissue levels of VEGF-A were elevated in both DSS and in TNBS models (data not shown), but that serum levels of VEGF-A were only significantly affected in TNBS colitis. Based on these findings we selected the TNBS model in BALB/c mice to give the highest possible contribution of VEGF-A towards gut inflammation as is suspected in human UC.

In the typical TNBS model of colitis, TNBS is dissolved in 20% ethanol, which destroys the epithelial layer allowing greater penetration of the TNBS hapten. However, we used 10% ethanol. Ethanol is known to induce many pro-angiogenic growth factors and receptors including VEGF, basic fibroblast growth factor (bFGF), transforming growth factor β (TGF-β), VEGF receptor 1 and VEGF receptor 2 resulting in angiogenesis not mediated by immune inflammatory mechanisms. Therefore, reducing the concentration of ethanol creates a more accurate representation of the role of VEGF-A in colitis [19]. Although the concentration of ethanol was lower in our model it was sufficient to disrupt the epithelial barrier and allow TNBS to initiate immune mediated inflammation and significant elevation of disease activity. Animals treated with rVEGF164_b_ were found to recover faster after each subsequent TNBS administration and maintain an average level of disease activity less than that of the untreated animals.

Our previous work has shown that rVEGF164_b_ inhibited endothelial proliferation and migration, two of the most important processes in new vessel formation during angiogenesis [14]. The rVEGF164_b_ blocked the expansion of the blood vascular system in the colon induced by TNBS treatment. However, there was still a modest increase in blood vessel density which may represent VEGF-A independent angiogenesis. We can infer the angiogenesis seen in our model is VEGF-A independent because the estimated level of rVEGF164_b_ expression that was detected far exceeded the level of VEGF-A in the serum. In addition, we have previously reported that rVEGF164_b_ can completely inhibit VEGF-A signaling at concentrations 10-fold lower than VEGF-A concentrations used in experiments [14].

One of the most interesting findings in this report was the reduction of the level of TNBS induced lymphangiogenesis by rVEGF164b administration is comparable to the reductions in angiogenesis. Lymphangiogenesis was increased in proportion to angiogenesis by TNBS which is in contrast to DSS colitis where levels of lymphangiogenesis are much higher than angiogenesis [20]. In fact, many reports have described the link between blood and lymphatic proliferation in several pathologies that have over expression of VEGF-A including cancer. While the exact relationship is unknown there are several possibilities for this observation [21-23]. First, VEGF-A over expression can drive the expression of VEGF-C and VEGF-D both of which are potent promoters of lymphangiogenesis resulting in functional vessels. Second, VEGF-A acting directly on lymphatic endothelium through VEGF receptor 2 can cause proliferation of lymphatic EC leading to overly large vessels that are deficient in pumping and result in edema. Third, VEGF-A can indirectly recruit lymphangiogenic lymphocytes through alterations of the blood vessels adhesive properties (CAM expression). Without further studies we cannot say with certainty what role rVEGF164_b_ plays in reducing lymphatic vessels. We are currently investigating these questions to determine any further potential benefits of rVEGF164_b_ therapy.

This study found that many of the markers of inflammation in UC (histopathology, neutrophil infiltration, follicle aggregate formation, colon length to weight ratio, etc.) were correlated with the level of angiogenic inhibition, and could be attenuated if not completely blocked by rVEGF164_b_ treatment. Disease activity and histopathology of rVEGF164_b_ treated animals revealed significantly less damage to the colon compared with untreated TNBS animals. Most of the decrease in DAI was due to reductions in occult blood and stool form. This result suggests there was less vascular leak due vessel permeability inhibition, reduced vessel damage, and less epithelial and mucosal damage, which cumulatively would affect these parameters. Of particular note to DAI is that at early stages (acute) of inflammation, DAI in treated and untreated animals receiving TNBS was statistically the same while other scores (MPO, follicle aggregate) were reduced. This may be due to exaggerated effects of mechanical damage by insertion of the enema needle on occult blood or anesthesia on weight loss, masking the effects of rVEGF164_b_ on DAI in the early stages of inflammation. Therefore, it may be more suitable to use the alternative methods of examining inflammation we employed to draw conclusions about the effects of rVEGF164_b_ on the acute phase of TNBS colitis. In this case, histopathology examination revealed that area involved, edema, inflammatory cell infiltration, and crypt loss were higher in untreated TNBS animals compared with rVEGF164_b_ treated animals at both 3 and 21 days. Although these individual scores were not significant individually, overall histopathology scores revealed a significant elevation in colon damage. Gross measurements of the colon revealed significant protection by rVEGF164_b_, which may be attributed to a lack of edema and reduced enlargement and contraction of the muscularis mucosae. The lack of edema may be directly linked to the anti-permeability as well as anti-angiogenic effects of rVEGF164_b_, permitting the lymphatic system to drain away excess fluid. Other factors may also be affected by rVEGF164_b_ in protection against colon shortening, suggesting that this anti-angiogenic VEGF has many extensive and unanticipated anti-inflammatory properties [16].

The rVEGF164_b_ treatment had a profound inhibitory effect on recruiting inflammatory cells to the colon in TNBS mice. This result may be due to reduced vessels/endothelial area, preservation of endothelial junction, and/or reduced adhesion molecule expression mediated by VEGF-A signaling [14,24-26]. MPO activity, a measurement of neutrophil infiltration into the tissue, was significantly reduced by rVEGF164_b_ in TNBS treated animals. This reduction was similar in magnitude to the decrease in angiogenesis and may represent reduction in VEGF-A activity on the endothelium. VEGF-A is one of the most important factors in IBD for recruiting neutrophils [24], the major source of MPO in tissues. Interestingly, MPO activity was significantly reduced at day 21 in rVEGF164_b_ treated animals despite the declining expression pattern of rVEGF164_b_ at this time point. In fact, the overall MPO score was much lower at day 21 than at day 3, even in animals treated with TNBS alone. This is likely because day 21 represents a late (recovery) phase of inflammation after the initial TNBS insult, in which most of the neutrophils have migrated away from the injured tissue. In addition, the rVEGF164_b_ treated animals showed reduced primary tissue injury leading up to the late (recovery) phase. Therefore these mice likely recovered from the injury faster than non-treated animals and would have subsequently less neutrophil infiltration.

Unlike many features of this model, the increase in follicle aggregates was completely blocked despite an incomplete blockade of angiogenesis. This could possibly be due a combination of reduction in the number of blood and lymph vessels and changes in the adhesive properties of the vessels, which mediate forming these structures. In addition, the formation of follicle aggregates heavily relies on recruiting macrophages to expand these structures, and VEGF-A is the most potent chemoattractant for this cell type [27,28]. This observation suggests that VEGF164_b_ may have many more beneficial mechanisms apart from those acting directly on the vasculature.

Many treatments of UC such as TNF-α inhibitory antibodies and mesalamine have indirect effects endothelial cells. Only one current treatment with natalizumab, addresses one of the many roles the microvasculature plays in UC [29-33]. The current standards for biological treatment of UC are TNF-α inhibitors (*e.g.*, Infliximab, Humera, *etc*.), which are effective clinically and in several animal UC models (including the TNBS model). It is difficult to compare the models of TNBS used in the studies of TNF inhibitors to our model given the adaptations that were used to reduce non-specific growth factor induction. However, it is interesting to note the trend of incomplete inhibition of disease activity was observed in TNBS mice treated with Infliximab, and many of the results that we obtained with rVEGF164_b_ treatment were similar.

## Conclusions

This is the first report using an endogenous inhibitory VEGF-A isoform in a model of experimental colitis. In this study we found the markers of UC disease showed improvements proportional to or greater than the level of angiogenic blockade that rVEGF164_b_ treatment provided. Our results confirm the pivotal role of the inflamed intestinal microvasculature in initiating and propagating gut inflammation. We also found that rVEGF164_b_ treatment significantly inhibited the level of lymphangiogenesis in our UC model. However, further studies are necessary to examine the role of rVEGF164_b_ on this component of UC involving changes in lymphatic proliferation, structure and function. Although we do not yet know how VEGF-A and VEGF164_b_ ratios change during active gut inflammation, VEGF164_b_ does appear to represent a new marker of disease with important bioactivity against VEGF-A mediated inflammation, angiogenesis (and lymphangiogenesis). VEGF164_b_ treatment has potential for further use investigating the role of these endogenous inhibitors of angiogenesis in UC. These results also show treatment with VEGF164_b_ as a promising new avenue for therapy in UC. Our future work will investigate potential VEGF164_b_ treatment in mice that already have developed TNBS-induced colitis.

## Abbreviations

UC: (Ulcerative colitis); VEGF: (Vascular endothelial growth factor); DAI: (Disease activity index); MPO: (Myeloperoxidase); CAM: (Cellular adhesion molecule); rVEGF: (Recombinant VEGF); Ad: (Adenovirus); bFGF: (Basic fibroblast growth factor); FGF: (Fibroblast growth factor).

## Competing interests

The authors declare that they have no competing interests.

## Authors’ contributions

WEC, JSA, and JMM conceived and designed the experimental approach. WEC, CVG, MP, and JT participated in the primary acquisition of the in vivo data. All authors contributed to the analysis and interpretation of the data. WEC, JSA, and JMM drafted and revised the manuscript. All authors read and approved the final manuscript.
